# Evaluating the satisfaction of patients utilising the virtual emergency department service in southeast region of Melbourne

**DOI:** 10.1111/1742-6723.70034

**Published:** 2025-04-02

**Authors:** Muhuntha Sri‐Ganeshan, Amanda Harris, Christopher Thuring, Gerard O'Reilly, Biswadev Mitra, Andrew Underhill, Claire Charteris, Diana Egerton‐Warburton, Peter A Cameron, the Southeast Region Virtual Emergency Department (SERVED) Research Group

**Affiliations:** ^1^ School of Public Health and Preventive Medicine Monash University Melbourne Victoria Australia; ^2^ Emergency and Trauma Centre The Alfred Hospital Melbourne Victoria Australia; ^3^ Emergency Department Monash Medical Centre Melbourne Victoria Australia; ^4^ School of Clinical Science at Monash Health Monash University Melbourne Victoria Australia

**Keywords:** emergency medical service, emergency medicine, patient satisfaction, telemedicine

## Abstract

**Objectives:**

Evaluate patient satisfaction with paramedic and residential aged care facility (RACF) staff‐initiated tele‐emergency care through the Southeast Melbourne Virtual ED.

**Methods:**

Patient satisfaction surveys were conducted across two of the constituent virtual EDs (VEDs).

**Results:**

Of 452 responses, 427 (94.5%) had no negative comments, with 341 (75.4%) rating the service 8 out of 10 or higher, and 83.4% would recommend the service to family or friends.

**Conclusions:**

Emergency telehealth services were favoured by patients, with ratings comparable to satisfaction scores in physical EDs. Upskilling of emergency clinicians for telehealth consultations and educational programmes could provide further improvements to patient experiences.

## Introduction

Tele‐emergency care (TEC) was initiated to re‐direct presentations away from the physical ED. Pilot studies, specifically examining paramedic‐initiated consultations (engaging a senior medical clinician) concluded that the technology could be utilised effectively to deliver TEC.^1^, ^2^ There has been an expansion of similar services across Australia despite questions remaining as to whether this is appropriate given the paucity of published research related to patient satisfaction. We examined patient perspectives as part of a routine 7‐day follow‐up, surveying a cohort of patients who utilised the Southeast Melbourne Virtual ED (SEMVED).

## Methods

SEMVED was a service covering Melbourne's southeast. Patients were referred by paramedics on scene or the staff of residential aged care facilities. Consultation occurred using patient or facility‐owned devices with patients assessed remotely by an emergency physician or general practitioner. Following review, if it was determined that alternative care pathways were more appropriate than ED, clinicians coordinated management *via* in‐person providers and provided medication for ongoing treatment by providing prescriptions. Further care was facilitated through referral to community treatment services, local medical practitioners, or direct inpatient admissions. Full details of the services have been previously reported.^3^

SEMVED involved three separate virtual EDs (VEDs) running concurrently, each run by the constituent health services. Two of these performed satisfaction surveys. These were performed independently by the health services for quality improvement, resulting in variation in implementation. Surveys were performed over the phone as part of routine follow‐up by care coordinators occurring 7 days post‐consultation for patients advised not to present to the physical ED. Feedback was from the patients themselves, except in cases where patients were unable to comply, in which case carers or residential aged care facility (RACF) staff completed the survey.

Surveys were performed between 8 January 2023 and 24 June 2023 for VED 1 and between 27 April 2022 and 17 July 2022 for VED 2. The survey utilised mixed‐methods questions adapted from Consumer Assessment of Healthcare Providers and Systems ED Survey. Questions related to patient experiences of staff interactions, their ability to comply with follow‐up plans and their global impression. Data were recorded predominantly utilising 3‐point Likert scales with the opportunity to provide additional comments. Comments were reviewed by two authors (AH, MS‐G) and key themes were identified. Surveys are included in Appendix S1 and S2.

### Ethical approval

Ethics approval for this project was obtained from the Alfred Health Human Research Ethics Committee (Project ID: 79/22) with site specific authorisation granted Monash and Peninsula Health.

## Results

**Figure 1:**
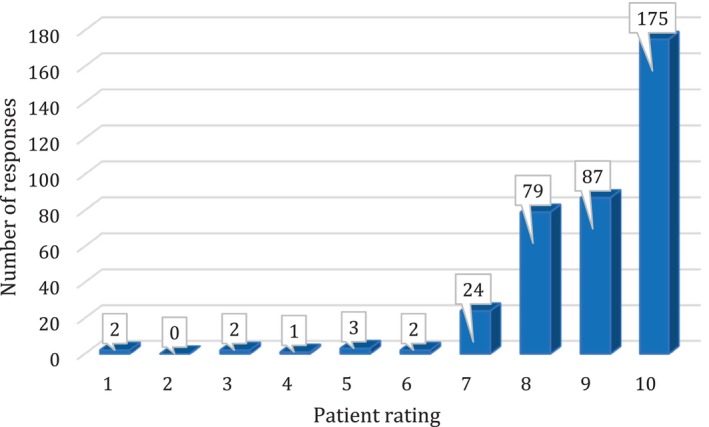
Overall rating of the VED service by patients. Higher rating indicated greater satisfaction; no rating provided by 77 respondents.

There were 223 responses from VED 1 (29.6% response rate) and 229 from VED 2 (32.3% response rate). Responses are outlined in Tables 1 and 2. The overall rating of the service is illustrated in Figure 1.

**TABLE 1:** Questionnaire responses: Patient satisfaction

	Yes	Neither Yes nor No	No	Total
Did the paramedics listen to and understand the patient's concerns?	387 (85.6%)	59 (13.1%)	6 (1.3%)	452 (100%)
Did the VED doctors listen to and understand the patient's concerns?	386 (85.4%)	59 (13.1%)	7 (1.5%)	452 (100%)
Was the VED follow‐up plan clearly explained to the patient?	406 (89.8%)	42 (9.3%)	4 (0.9%)	452 (100%)
Would the patient use the VED again or recommend it to family/friends?	377 (83.4%)	67 (14.8%)	8 (1.8%)	452 (100%)

**TABLE 2:** Questionnaire responses: Patient outcomes

	Yes	Neither Yes nor No	No	Total
Did the patient access additional forms of healthcare outside of their VED follow‐up plan?	202 (44.7%)	65 (14.4%)	185 (40.9%)	452 (100%)
Did the patient follow the treatment advice they were given?	424 (93.8%)	24 (5.3%)	4 (0.9%)	452 (100%)

	Improved	Neither Improved nor Deteriorated	Deteriorated	Total
Did the patient's condition improve, deteriorate, or remain the same?	397 (87.8%)	47 (10.4%)	8 (1.8%)	452 (100%)

Based on comments from VED 1, negative feedback was reported by three patients about paramedics and three about the VED clinician. From VED 2, 12 patients left negative comments and seven left mixed comments. These largely centred around displeasure using an online platform (*n* = 5) and poor communication (*n* = 5). These patients stated that the clinicians ‘didn't really listen’ or that ‘no one [was] prepared to answer [their] questions’. Some patients (*n* = 4) had difficulties receiving medication or medical certificates after the VED consultation and noted (*n* = 1) that there was ‘no contact point to address [the] doctor with this issue’. Additionally, connectivity issues (*n* = 3) prevented patients from being able to understand and communicate effectively with the clinician.

## Discussion

The SEMVED provided a high‐quality consumer experience. Our results compare favourably to physical ED presentations, with The Australian Bureau of Statistics reporting that only 64.6% of patients felt they had been listened to by doctors in the ED.^4^ Results are consistent with other studies looking into alternate models of TEC, with Reid *et al*. reporting that from a cohort of patients utilising a paediatric virtual ED, 87% of respondents rated the service as nine or 10 out of 10.^5^

The study is limited by a low response rate, with a potential for those not responding having adverse outcomes. It does, however, provide insight as to how the service might be improved. Beyond some logistical considerations, feedback suggests that the communication skills required for conducting a telehealth consultation differ from those required conventionally. This was a novel modality for patients and may be limited in ability to express concerns as well as fully understand what was intended by the clinician. A satisfactory consultation likely requires clinician experience or specific training. The use of data linkage to gain insight into the patient journey and its influence on patient satisfaction would be of particular benefit and is a consideration for future research.

TEC consultations provided a high‐quality consumer experience for most. Ongoing delivery of such services would benefit from further analysis of patient and carer experiences.

## Supporting information


**Appendix S1.** Alfred Questionnaire.
**Appendix S2.** Monash Questionnaire.

## Data Availability

The data that support the findings of this study are available on request from the corresponding author. The data are not publicly available due to privacy or ethical restrictions.
