# Supplementary material for: Evaluating the satisfaction of patients utilising the virtual emergency department service in southeast region of Melbourne
Source: Emerg Med Australas. 2025 Apr 2;37(2):e70034. doi: 10.1111/1742-6723.70034 (PMC11963220; doi:10.1111/1742-6723.70034)
Supplement: Supplementary file 1 — Appendix S1. Alfred Questionnaire. Appendix S2. Monash Questionnaire. [file EMM-37-0-s001.docx]

**Appendix S1: Alfred Questionnaire**

1. Did you feel your concerns were listened to and understood by the Paramedic?

- Agree
- Disagree
- Neither
- Free text for specific comments

1. Did you feel your concerns were listened to and understood by the Emergency Doctor?

- Agree
- Disagree
- Neither
- Free text for specific comments

1. Was your follow-up plan from the Virtual ED telehealth consult explained in a way you could understand?

- Agree
- Disagree
- Neither
- Free text for specific comments

1. Were you able to complete/adhere to the treatment advice?

- Agree
- Disagree
- Neither
- Free text for specific comments

1. Did you use any additional health care that was outside of your follow up plan?

- Recalled an ambulance
- GP /doctor appointment
- Local pharmacist
- Attended an emergency department – were they admitted?
- I did not seek further help
- Other- please specify

1. Has your condition:

- Improved
- Deteriorated
- Stayed the same

1. Overall on a scale of 1-10, how would you rate your experience using virtual ED?

- Rating 1-10

1. Would you use the Virtual ED again and/or recommend it to family/friends?

- Yes
- No
- Maybe

1. Would you be willing to be contacted to provide additional feedback on your experience using the Virtual ED?

- Yes
- No
- Maybe

1. Are there any other comments you would like to make about your Virtual ED experience?

**Appendix S2: Monash Questionnaire**

1)The paramedic listened to and understood my concerns

- Agree
- Disagree
- Cannot comment

2) The Virtual ED doctor listened to and understood my concerns

- Agree
- Disagree
- Cannot Comment

3)The follow up plan was clearly explained to me.

- Agree
- Disagree
- Cannot Comment

4) I have followed the treatment advice given.

- Agree
- Disagree
- Cannot Comment

5)Did you use any additional health care that was outside of your follow up plan?

- Recalled an ambulance
- GP /doctor appointment
- Local pharmacist
- Attended an emergency department – were they admitted?
- I did not seek further help
- Other- please specify

1. Has your condition improved, deteriorated or stayed the same?:

- Improved
- Deteriorated
- Stayed the same

1. Overall on a scale of 1-10, how would you rate your experience using virtual ED?

- Rating 1-10

1. Would you use the Virtual ED again and/or recommend it to family/friends?

- Yes
- No
- Maybe

1. Would you be willing to be contacted to provide additional feedback on your experience using the Virtual ED?

- Yes
- No
- Maybe

1. Are there any other comments you would like to make about your Virtual ED experience?
